# The truncated mutant HBsAg expression increases the tumorigenesis of hepatitis B virus by regulating TGF-β/Smad signaling pathway

**DOI:** 10.1186/s12985-018-0972-0

**Published:** 2018-04-02

**Authors:** Meng-Lan Wang, Dong-Bo Wu, Ya-Chao Tao, Lan-Lan Chen, Cui-Ping Liu, En-Qiang Chen, Hong Tang

**Affiliations:** Center of Infectious Diseases, West China Hospital, Sichuan University, No.37 Guo Xue Xiang, Wuhou District, Chengdu, 610041 People’s Republic of China

**Keywords:** Hepatitis B virus mutation, rtA181T/sW172* mutation, Truncated mutant HBsAg, Tumorigenises, TGFBI

## Abstract

**Background:**

It has been reported that the emergence of HBV rtA181T/sW172* mutant could result in a dominant secretion defect of HBsAg and increase the risk of HCC development. This study was designed to reveal the role and possible pathogenic mechanism of truncated mutant HBsAg in tumorigenesis of HBV rtA181T/sW172* mutant.

**Results:**

As compared to wide type or substituted mutant HBsAg, the ratio of cell clones was significant higher in L02 cells stable expressing truncated mutant HBsAg. Injection of L02 cells stable expressing truncated mutant HBsAg into the dorsal skin fold of nude mice resulted in increased primary tumor growth compared to L02 cells stable expressing wide-type and substituted mutant HBsAg. In HBV replication L02 cell lines, the key molecular involved in TGF-β/Smad pathway was also investigated. We found that the mRNA and protein levels of Smad3/2, CREB and CyclinD1 were significantly higher and TGFBI level was significantly lower in cells stably expressing truncated mutant HBsAg as compared to cells stably expressing wide-type and substituted mutant HBsAg. Additionally, after administration of TGF-β1 (increasing TGFBI level), the volume of tumor is obviously reduced in nude mice with injection of L02 cells stable expressing truncated HBsAg.

**Conclusions:**

The emergence of sW172* mutant may increase the tumorigenesis of HBV, and its mechanism may be associated with down-regulated expression of TGFBI in TGF-β/Smad signaling pathway.

## Background

Hepatitis B virus (HBV) chronic infection is the key cause of hepatocellular carcinoma (HCC) in Asians. Oral nucleos(t)ide analogs (NAs) have been proven to be effective in suppressing HBV replication, decreasing liver inflammation and fibrosis, and reducing the risk of HCC development [1]. Because all available NAs selectively target the reverse transcriptase (RT) domain of HBV DNA polymerase, mutations in HBV-RT region were naturally screened during the long term use of NAs [2]. The emergence of these mutants would not only inevitably lead to viral breakthrough and hepatic dysfunction, but also influence the biological characteristics and pathogenicity of HBV, which may increase risk of disease progression and HCC occurrence [3, 4].

In past decades, lamivudine and adefovir dipivoxil has been widely used in the treatment of patients with CHB. However, because of a low genetic barrier to the development of resistance, HBV rtA181T/V mutation is common in real-life medical practice [2]. As we known, HBV rtA181T mutant is an A → T mutation at position 181 of the P gene in the RT domain. In theory, its overlapping S gene should have two types of mutation at amino acid 172, which include rtA181T/sW172L (TGG CTC → TTA CTC) mutant coding substituted HBsAg protein and rtA181T/sW172*(TGG CTC → TGA CTC) mutant coding truncated HBsAg protein [5]. Our previously studies showed that the replication capacity of HBV rtA181T/sW172* mutant was higher than that of HBV rtA181T/sW172L mutant [6]. HBV rtA181T/sW172* mutant had a dominant secretion defect of HBsAg which may cause the accumulation of viral core particles in liver cells, and the latter may damage the liver microenvironment [6, 7]. Additionally, clinical study indicated that HBV carrying rtA181T/sW172* mutation may have an increased oncogenic potential [8]. However, it is still unknown about the molecular mechanism of tumorigenesis of HBV rtA181T/sW172* mutant expressing truncated HBsAg protein.

Thus, to better and fully understand the tumorigenicity of HBV rtA181T mutants, we designed this study to investigate the impact of different overlapping S gene mutation (sW172L and sW172*) on the tumorigenicity of HBV rtA181T mutant and reveal the possible mechanism.

## Methods

### Study design

This study obtained ethics approval from the Laboratory Animal Ethics Committee of Sichuan University, and all animal experiments were performed in accordance with relevant guidelines and regulations. Present experiment consisted of two parts. The first part was to investigate and compare the tumorigenesis of different overlapping S gene mutation (sW172L and sW172*) on rtA181T mutant strain in vitro and in vivo. The second part was to investigate the possible molecular signaling pathway associated with tumorigenises of truncated mutant HBsAg in cells in vitro and nude mice in vivo.

### Cell, animal and plasmid used in this study

Normal liver cell (L02) lines were conserved in our laboratory. They were grown in RPMI 1640 Medium (Invitrogen) supplemented with 10% fetal bovine serum, 100 U/mL penicillin, and 100 μg/mL streptomycin; and cultured at 37 °C in a humidified incubator with 5%CO_2_. Female BALB/c nude mice (3 weeks old, weighing 10 to 12 g) were obtained from Laboratory Animal Center of Sichuan University (Chengdu, China) and raise in SPF environment at room temperature.

The wild-type pHBV4.1-HBs(wt), pHBV4.1-HBs(sW172L) and pHBV4.1-HBs(sW172*) mutant plasmids were previously constructed. The wild-type pHBV4.1 is an HBV replication competent plasmid, which contains 1.3 copies of HBV genome (subtype ayw) and capable of process complete replication both in vitro and in vivo. The pHBV4.1-HBs(sW172L) and pHBV4.1-HBs(sW172*) mutant plasmids were generated using wild-type pHBV4.1 as the template. Plasmid pHBV4.1-HBs(sW172L) could express substituted HBsAg, while pHBV4.1-HBs(sW172*) could express truncated HBsAg. Additionally, the wild-type, substituted and truncated HBsAg eukaryotic expression plasmids of pcDNA3.1-HBs(sW172L), pcDNA3.1-HBs(sW172*) and pcDNA3.1-HBs(wt) were also constructed and conserved in our laboratory.

In present study, stably transformed L02 cell lines with persistent expression of wild-type HBsAg (pcDNA3.1-HBs(wt) and pHBV4.1-HBs(wt)), substituted mutant HBsAg (pcDNA3.1-HBs(sW172L) and pHBV4.1-HBs(sW172L)), and truncated mutant HBsAg (pcDNA3.1-HBs(sW172*) and pHBV4.1-HBs(sW172*)) were also established and used in vitro and in vivo experiments.

### Soft agar colony formation experiment

Colony formation and cell growth rate in soft agar were tested by plating 2 × 10^3^ L02 cells stably transfected with plasmid, supplemented with 100 units/mL penicillin, 100 μg/mL streptomycin, 10% fetal bovine serum, and 0.7% low melting temperature agarose in 6-well plates (3 wells for each) coated with 1.5 mL of 1.3% low melting temperature agarose. Colony formation of each type of plasmid-transfected cells was monitored for 21 days in 37 °C incubator, and colony number and size was recorded for comparison and then microphotographed by optical microscope.

### Nude mouse tumorigenicity experiment

BALB/c nude mice received subcutaneous inoculation in the dorsal skin fold of 1 × 10^5^ plasmid-transfected L02 cells suspended in 100 μL of cold PBS. The cells were growing at 40–50% confluence before collection. Additionally, some of above BALB/c nude mice were also given TGFβ1(Peprotech, UK) at the dose of 2 ng/ml·20 g via tail vein injection to clarify whether TGF-β/Smad pathway was involved in the tumorigenicity of HBV-rtA181T/sW172* mutant.

In nude mouse tumorigenicity experiment, the observation period was 13 weeks, and tumor size was measured every week. Mice were sacrificed at week 13 and transplantable tumors were excised and processed for hematoxylin-eosin(HE) staining. Tumor length and width were measured using calipers, and tumor volume was calculated as π/6 × length×width×width.

### Western blotting analysis

The protein expression levels of molecules involved in tumor-related signaling pathways in L02 cells were determined by western blot analysis, according to the standard manufacturer’s protocol. And the antibodies of mouse monoclonal TGFBI, rabbit polyclonal CREB, rabbit polyclonal Smad2/3 and rabbit polyclonal Cyclin D1 were all purchased from Cell Signaling Technology, Inc. (Beverly, MA, USA); and the antibody of mouse polyclonal GAPDH was purchased from Jackson ImmunoResearch Laboratories (West Grove, PA, USA). The antibodies of TGFBI, CREB, Smad2/3, Cyclin D1 and GAPDH were all diluted at 1: 1000; while the secondary antibody was diluted at 1: 3000. Immunodetection was performed with the ECL-Plus kit (Pierce Biotechnology, USA), and immunoblot signals were quantified using Quantity One Software (Bio-Rad).

### Real-time quantitative PCR analysis

Total RNA was extracted from L02 cells with TRIzol Reagent (Invitrogen, USA). Total cellular RNA was reverse-transcribed using Moloney Murine Leukemia Virus (MMLV) reverse transcriptase (Gibco BRL, USA). Blank reactions with no RNA were performed in all experiments. The expression of genes mRNA was measured by real-time PCR using Maxima SYBR green/ROX qPCR Master Mix (Fermentas Life Sciences, Canada). The primer sequences a of GAPDH (the reference) and candidate genes are listed in Table 1. And the fold changes of the expression of the candidate genes relative to the reference gene were calculated using the normalized expression (ΔCt) method with default threshold values using CFX Manager Software (Bio-Rad).

**Table 1:** Primer sequences used in the quantitative RT-PCR analysis

Gene	Forward Primers	Reverse Primers
AP-1	ACGCAAACCTCAGCAACTTCA	GATCCGCTCCTGGGACTCCAT
c-Myc	GTTTCATCTGCGACCCG	GCTGCCGCTGTCTTTGC
c-Fos	TCCGAAGGGAAAGGAATAA	GCTGCCAGGATGAACTCTA
CyclinD1	ACTCGTTACATACCCTTTACCTTTT	TAACTCTGATAGACTTTTGCCATTC
NF-kBp65	TGCCGAGTGAACCGAAAC	TGGAGACACGCACAGGAG
TGFBI	CTTCGAGAAAGATCCCTAGTGAGA	CGTTGATAGTGAGCATGTCCC
GAPDH	AGGAGCGAGATCCCTCCAAAATCAAGT	TGAGTCCTTCCACGATACCAAAGTTGT

## Results

### Truncated HBsAg induces tumorigenesis in L02 cells and nude mice

**Fig. 1:**
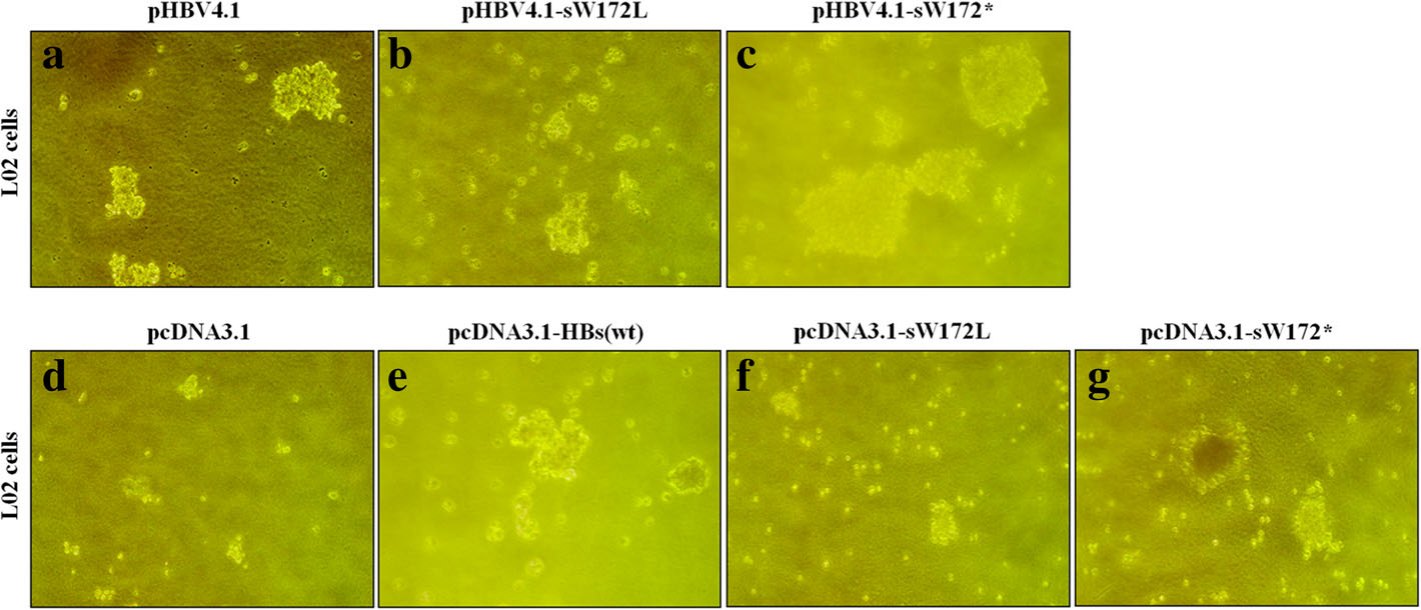
Formation of colonies in soft agar. The ratio of cell clones were less than 30% in L02-pHBV4.1-HBs(wt) (**a**) and L02-pHBV4.1-HBs(sW172L) (**b**), but well formed with a ratio of 60% in L02-pHBV4.1-HBs(sW172*) cell clones (**c**). Though the ratio of cell clones were less than 20% in either L02-pcDNA3.1 (**d**), L02-pcDNA-HBs(wt) (**e**), L02-pcDNA-HBs(sW172L) (**f**) and L02-pcDNA-HBs(sW172*) (**g**), the ratio of cell clones was more in L02-pcDNA-HBs(sW172*) than in other three groups

In this study, tumor-forming ability of truncated HBsAg was determined using soft agar colony-forming assay in vitro and nude mice xenografts in vivo. After a cultural period of 21 days in soft agar, L02-pHBV4.1-HBs(sW172*) cell clones were well formed with a ratio of 60%; while the ratio of cell clones were less than 30% in L02-pHBV4.1-HBs(sW172L) and L02- pHBV4.1-HBs(wt). Though the ratio of cell clones were less than 20% in either L02-pcDNA3.1, L02-pcDNA3.1-HBs(wt), L02-pcDNA3.1-HBs(sW172L) or L02-pcDNA3.1-HBs(sW172*), the ratio of cell clones was more in L02-pcDNA3.1-HBs(sW172*) than other three groups (Fig. 1).

**Fig. 2:**
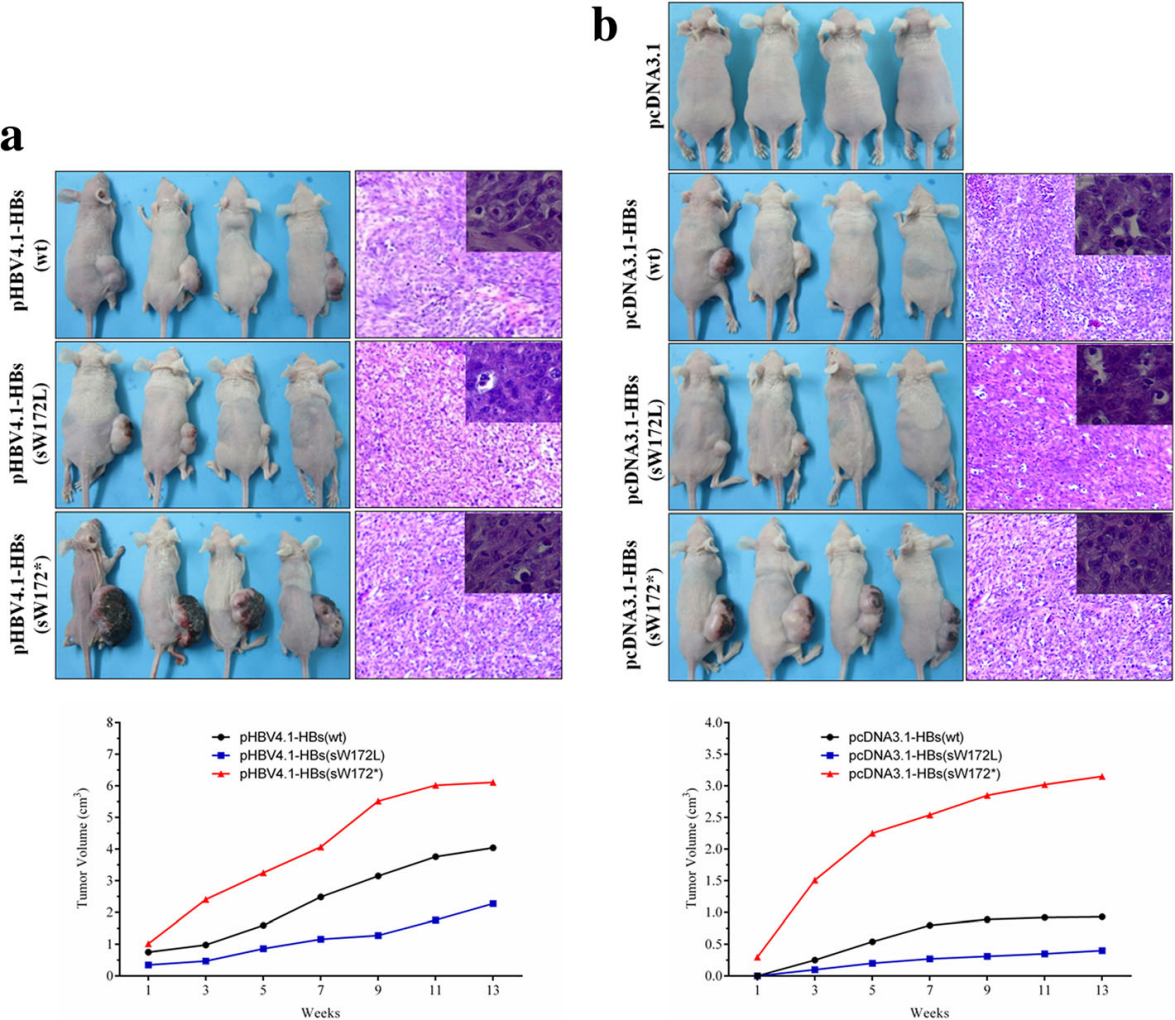
The gross pathological and histopathological changes of xenografts in nude mouse. **a** Nude mice injected with HBV replication L02 cell lines stably expressing wild type, substitute and truncated HBsAg, respectively; (**b**) nude mice injected with L02 cell lines stably expressing wild type, substitute and truncated HBsAg, respectively. The tumor volume was significantly higher in nude mice with pHBV4.1-HBs(sW172*) or pcDNA3.1-HBs(sW172*) as compared to other groups

The gross pathological and histopathological changes of xenografts in nude mouse were shown in Fig. 2a and b. Among nude mice injected with HBV replication L02 cell lines stably expressing HBsAg, the tumorgenesis rates of pHBV4.1-HBs(wt), pHBV4.1-HBs(sW172L) and pHBV4.1-HBs(sW172*) group were all 100% (4/4). However, as compared to nude mice with pHBV4.1-HBs(wt) and pHBV4.1-HBs(sW172L), the tumor volume was significantly higher in nude mice with pHBV4.1-HBs(sW172*) (Fig. 2a).

Among nude mice injected with L02 cell lines stably expressing HBsAg(without HBV replication), the tumorgenesis rate of pcDNA3.1(blank control), pcDNA3.1-HBs(wt), pcDNA3.1-HBs(sW172L) and pcDNA3.1-HBs(sW172*) group were 0 (0/4), 50%(2/4), 50%(2/4) and 100% (4/4), respectively. Additionally, the tumor volume was significantly larger in nude mice with pcDNA3.1-HBs(sW172*) as compared to other three groups (Fig. 2b).

### Truncated HBsAg regulates key molecules expression involved in TGF-β/Smad pathways

**Fig. 3:**
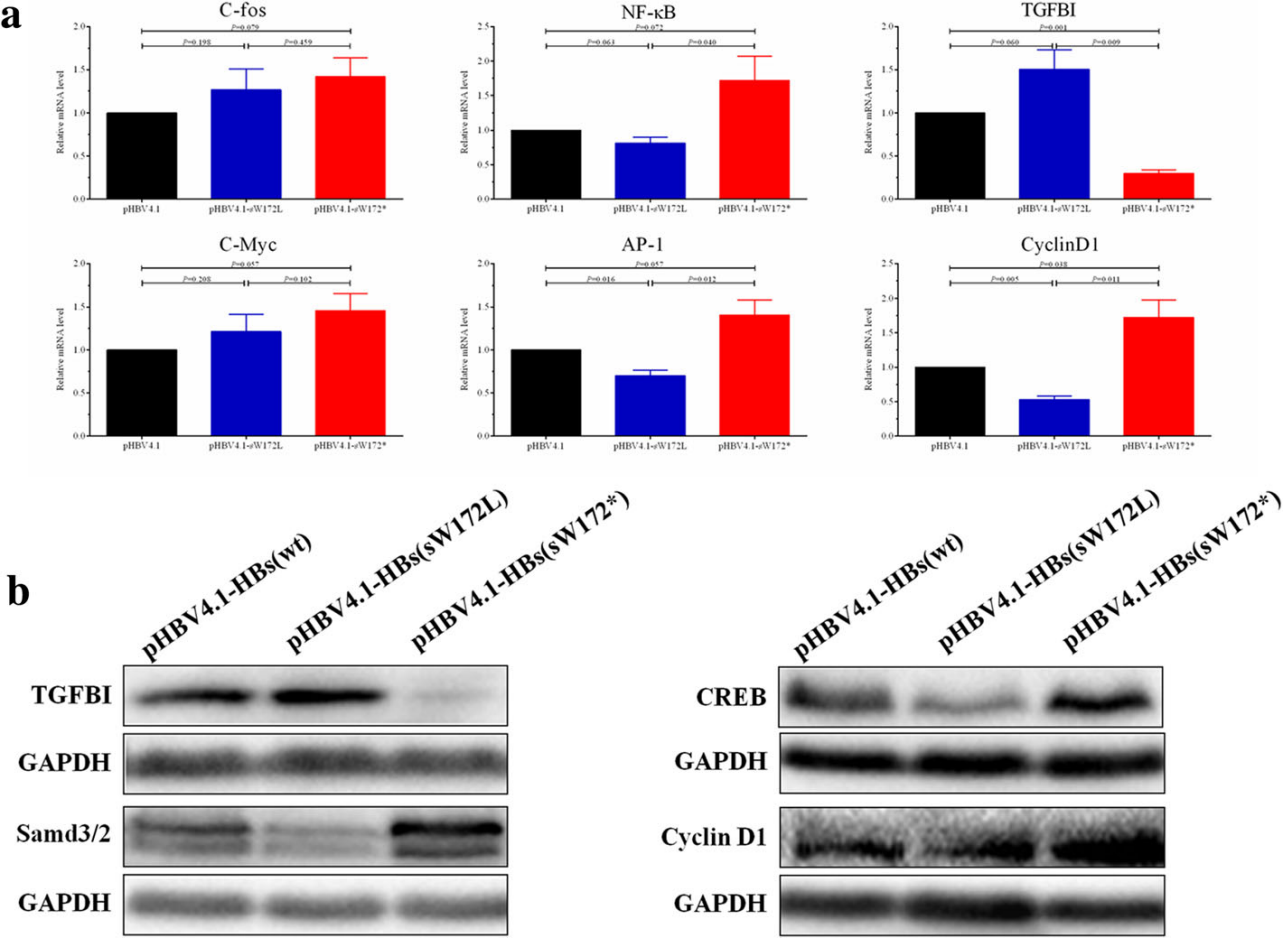
HBV rtA181T/sW172* mutant regulates key molecules expression involved in TGF-β/Smad pathways. **a** Relative mRNA levels of molecules involved in TGF-β/Smad (TGFBI and CyclinD1) pathway as well as LEF/Wnt (C-Myc and C-fos) and c-Raf-1/Erk2 (AP-1 and NF-kappaB) pathways; (**b**) the protein levels of TGFBI, Samd3/2, CREB and CyclinD1 involved in TGF-β/Smad pathways

In this study, the mRNA levels of molecules involved in LEF/Wnt (C-Myc and C-fos), c-Raf-1/Erk2 (AP-1 and NF-kappaB) and TGF-β/Smad (TGFBI and CyclinD1) pathways were measured in pHBV4.1-HBs(wt), pHBV4.1-HBs(sW172L) and pHBV4.1-HBs(sW172*) stably transfected L02 cells, respectively (Fig. 3a). As compared to pHBV4.1-HBs(wt) and pHBV4.1-HBs(sW172L) transfected cells, the mRNA levels of TGFBI significantly decreased and CyclinD1 significantly increased in pHBV4.1-HBs(sW172*) transfected cells. But the difference in mRNA levels of C-Myc, C-fos, AP-1 and NF-kappaB were not significant among three groups.

Additionally, we also examined the protein expression of key molecules relevant to TGF-β/Smad pathway. As compared to pHBV4.1-HBs(wt) and pHBV4.1-HBs(sW172L) transfected cells, the protein levels of Smad3/2, CREB and CyclinD1 were significantly higher and TGFBI level was significantly lower in pHBV4.1-HBs(sW172*) transfected cells (Fig. 3b).

### Increasing TGFBI expression inhibits the growth of tumor induced by pHBV4.1-HBs(sW172*) in nude mouse

**Fig. 4:**
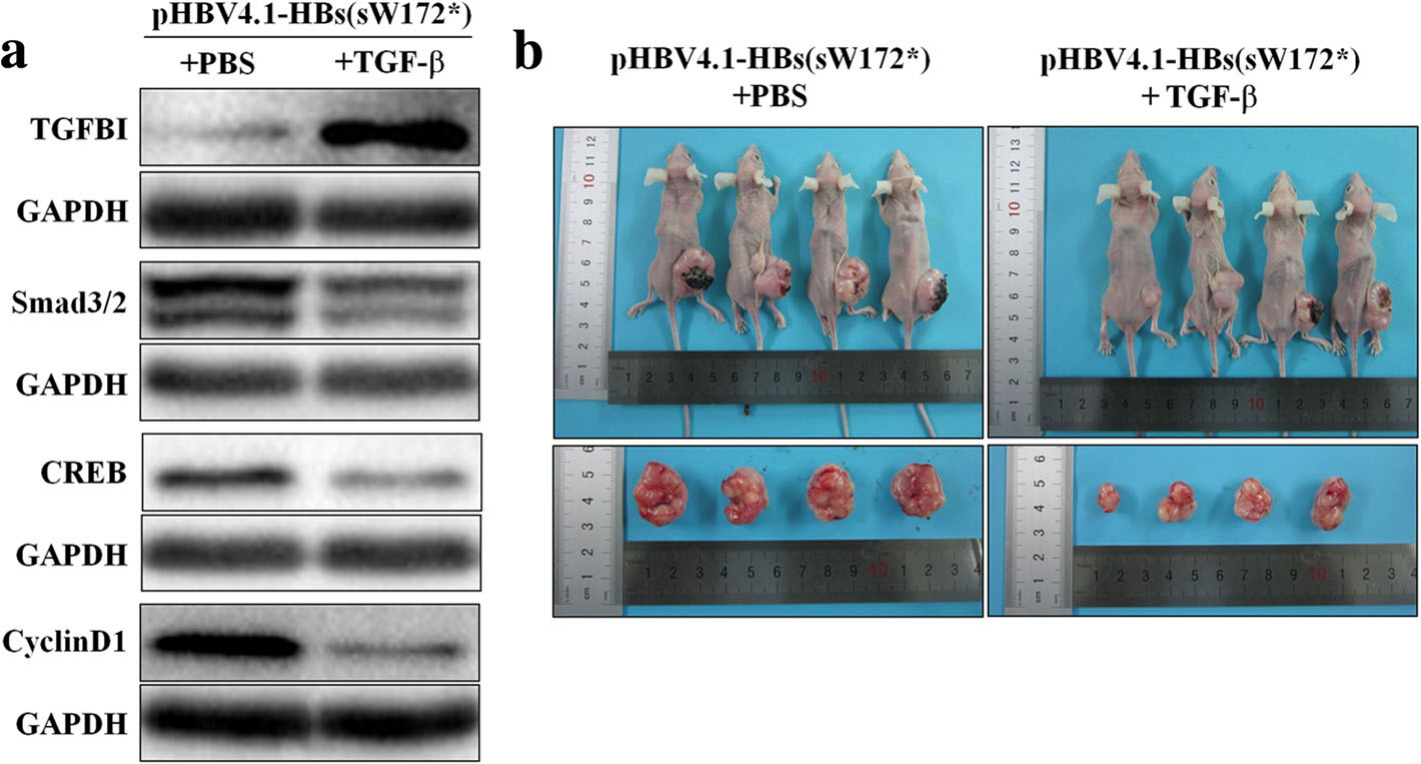
Increasing TGFBI expression inhibits the growth of tumor in nude mouse. **a** Protein levels of TGFBI, Samd3/2, CREB and CyclinD1 involved in TGF-β/Smad pathway; (**b**) tumor size in nude mouse

Considering that TGFBI is located upstream of TGF-β/Smad, we further increased the protein expression of TGFBI by administration of TGF-β in vitro and in vivo. In L02 cells with stably transfected pHBV-HBs(sW172*), the levels of Smad3/2, CREB and CyclinD1 decreased significantly after the administration of TGF-β1. Importantly**,** the tumor size of nude mice transplanted with stably transfected pHBV4.1-HBs(sW172*) L02 cells were significantly smaller in TGF-β1 treatment group than in the PBS group (2.33±0.79 cm^3^ vs.4.45±1.03 cm^3^, *P* = 0.017) (Fig. 4).

## Discussion

Though HBV belongs to hepatotropic DNA virus family, its pathogenic mechanism is not directly damaging to liver cells. Instead, host cellular immunity and humoral immunity against HBV should be the main reason causing liver damage. It has been reported that the HBsAg produced by some HBV mutants may help virus particles escaping immune surveillance, thus hindering the body’s immune system killing HBV, leading to persistent infection of HBV and continuous liver injury, and finally resulting in the development of liver cirrhosis or even HCC [9, 10]. Additionally, HBsAg protein can interfere with some important signaling pathways in hepatocyte, which may induce malignant proliferation of hepatocytes and lead to the occurrence of hepatocellular carcinoma [4, 11]. In present study, because of the deletion of 55 amino acids at the carboxyl terminus of HBsAg, the spatial structure HBsAg expressed by HBV rtA181T/sW172* has been changed, and this may be the molecular basis for enhanced pathogenicity of HBV rtA181T/sW172* mutant.

Currently, both soft-agar colony forming assay and nude mouse tumorigenesis test have been recommended by WHO and US FDA for tumorigenicity evaluation. In present study, the results of above two experiments both suggested an increased tumorigenic ability of L02 cells expressing truncated mutant HBsAg as compared to that of L02 cells expressing substituted or wide-type HBsAg. And this finding is also consistent with the clinical increased risk of developing HCC in CHB patients with evidence of HBV rtA181T/sW172* mutant [8, 12]. In present study, as compared to L02 cell lines just expressing HBsAg proteins, the larger size and faster formation of cell mass and transplanted tumor in L02 cell lines expressing whole genome of HBV suggested that there was a synergistic effect of truncated mutant HBsAg in tumorigenicity with coding genes in other regions of HBV.

In our previous study, we found that the viral replication in pHBV4.1-HBs(sW172*) mutant increased and maintained significantly longer than that in pHBV4.1-HBs(sW172L) mutant, and truncated mutant HBsAg also could increase the activity of HBV core promoter which could further increase transcription and replication of HBV genome [6, 7]. As we known, active HBV replication is associated with the high risk of HCC development [13]. Thus the enhancement of HBV replication mediated by truncated mutant HBsAg may be involved in the pathogenicity enhancement of HBV.

In present study, we also investigated the molecular mechanism of tumorigenesis of truncated mutant HBsAg using L02 cells with stably transfected pHBV4.1-HBs(sW172*), and L02 cells with stably transfected pHBV-HBs(sW172L) and pHBV4.1-HBs(wt) were used as control. The key molecules involving in TGF-β/Smad pathway were significantly changed, with low expression of anti-tumor factor TGFBI and high expression of CyclinD1(oncogene) in downstream of TGFBI. And the trends of TGFBI, CREB, Smad2, Smad3 and CyclinD1 expression in L02 cells with truncated, substitute and wide-type HBsAg stable expression were all consistent with corresponding finding of soft-agar colony forming assay and nude mouse tumorigenesis experiment. And the difference in viral replication capacity of pHBV4.1-HBs(wt), pHBV4.1-HBs(sW172L) and pHBV4.1-HBs(sW172*) should be an important cause of different expression of Smad3/2 and CREB in present study.

It is well-known that CyclinD1 is an important regulator of host cell cycle, and it could promote cells from G1 phase to S phase that would shorten the period of cell cycle and accelerate cell proliferation. Conversely, the decreasing of CyclinD1would result in a reduction of cell proliferation but an increasing of cell apoptosis. Evidences have shown that excessive promotion of cell proliferation play important roles in cell malignant transformation. In fact, we also found that the proliferation of L02 cells was significantly increased in the presence of truncated mutant HBsAg (data unshown). TGFBI(transforming growth factor, β-induced), also known as βig-H3, is a protein induced by TGFβ and found in a wide variety of tissues. It conduces to cell adhesion by interactions with integrins as same as many parts of the extracellular matrix (ECM). There are quite a few causative evidences revealing either the negative or positive role of TGFBI playing in tumorigenesis. TGFBI functions as a tumor suppressor has been found in many tumors, as in human lung carcinoma, ovarian carcinoma and breast carcinoma [14, 15]. In present study, after restoration of TGFBI expression in L02 cells with stably transfected pHBV4.1-HBs(sW172*), the tumor volume was significantly reduced in nude mice; and the expression of CyclinD1 decreased in L02 cells accompanied by a decreasing of cell proliferation and increasing of cell apoptosis(data unshown). Thus, we speculated that the pathogenic mechanism of truncated mutant HBsAg inducing tumorigenesis may be associated with TGF-β/Smad pathway via regulating cell cycle. In fact, we also have other evidences in support of this speculation. For example, Porf. Locarnini et al. has been reported that the presence of HBV rtV191I/sW182* mutant may induce cell canceration via inhibiting TGFBI expression and increasing CyclinD1expression [16]. And Porf. Lee et al.found that the percentage of S-phase cells increased in the presence of HBV rtV191I/sW182* as compared to that in the presence of wide-type HBV, and the possible mechanism may be associated with the down-regulation of p53 and p21 and up-regulation of Cyclin A and CDK4 gene expressions [17], which also could promote cells from G1 phase to S phase thus accelerating cell proliferation.

In present study, there was no significant pathogenicity enhancement of sW172L mutation as compared to sW172*. The main reason may be associated with spatial structure difference between substituted mutant HBsAg and truncated mutant HBsAg. As we known, truncated mutant HBsAg lost the C-terminal membrane translocation signal because of the C-terminal large deletions, and this change of spatial structure would impair the secretion of truncated mutant HBsAg and result in accumulation of truncated mutant HBsAg in cytoplasm. As a product of pre-S2 /S gene with at least 50 amino acids deletion in C-terminal, truncated mutant HBsAg is also reported to be a trans activator that could induce activation of a large number of oncogene via combining with PKC and regulating transcription factors such as AP-2. Additionally, truncated mutant HBsAg is also could directly regulate the activation of some gene promoters [18]. Thus, the transcriptional activation of truncated mutant HBsAg may be related to the increased possibility of tumorigenesis in HBV-rtA181T/sW172* mutant.

In present study, we also investigated the molecular mechanism of tumorigenesis of truncated mutant HBsAg using L02 cells with stably transfected pHBV4.1-HBs(sW172*), and L02 cells with stably transfected pHBV4.1-HBs (sW172L) and pHBV4.1-HBs(wt) were used as control. Though the changes of key molecules in LEF/Wnt pathway and proteinase C (PKC)-dependent c-Raf-1/Erk2 pathway were not significant than key molecules in TGF-β/Smad pathway, we could not completely deny the possible role of LEF/Wnt and PKC- dependent c-Raf-1/Erk2 pathways in the tumorigenic mechanism of HBV-sW172* truncated mutant, and further research is still needed to clarify this issue.

## Conclusion

The emergence of sW172* mutant coding truncated mutant HBsAg may increase possibility of HBV tumorigenesis, and its mechanism may be associated with down-regulated expression of TGFBI in TGF-β/Smad signaling pathway. However, the exact biological characteristics of HBV mutants coding truncated mutant HBsAg is still unclear and the exact mechanism of truncated mutant HBsAg increasing the tumorigenesis of HBV is still need more studies to reveal.

## Abbreviations

HBsAg: Hepatitis B surface antigen
HBV: Hepatitis B virus
HCC: Hepatocellular carcinoma
NAs: Nucleos(t)ide analogs
RT: Reverse transcriptase
TGFBI: Transforming growth factor beta-induced
TGF-β: Transforming growth factor-β
